# Genetic Basis for Resistance Against Viral Nervous Necrosis: GWAS and Potential of Genomic Prediction Explored in Farmed European Sea Bass (*Dicentrarchus labrax*)

**DOI:** 10.3389/fgene.2022.804584

**Published:** 2022-03-25

**Authors:** Sergio Vela-Avitúa, Ingunn Thorland, Vasileios Bakopoulos, Kantham Papanna, Arkadios Dimitroglou, Eleftherios Kottaras, Papaharisis Leonidas, Bruno Guinand, Costas S. Tsigenopoulos, Muhammad L. Aslam

**Affiliations:** ^1^ Benchmark Genetics Norway AS (formerly Akvaforsk Genetics Center AS), Sunndalsøra, Norway; ^2^ Laboratory of Ichthyology, Aquaculture and Diseases of Aquatic Animals, Department of Marine Sciences, University of The Aegean, Mytilene, Greece; ^3^ Nireus Aquaculture SA., Koropi, Greece; ^4^ CNRS, IRD, EPHE, ISEM, Université de Montpellier, Montpellier, France; ^5^ Biotechnology and Aquaculture (IMBBC), Hellenic Centre for Marine Research (HCMR), Institute of Marine Biology, Heraklion, Greece; ^6^ Nofima AS, Tromsø, Norway

**Keywords:** nervous necrosis virus, viral nervous necrosis, VNN, single nucleotide polymorphisms, genomic prediction, quantitative trait loci, genome-wide association analysis, heritability

## Abstract

Viral nervous necrosis (VNN) is an infectious disease caused by the red-spotted grouper nervous necrosis virus (RGNNV) in European sea bass and is considered a serious concern for the aquaculture industry with fry and juveniles being highly susceptible. To understand the genetic basis for resistance against VNN, a survival phenotype through the challenge test against the RGNNV was recorded in populations from multiple year classes (YC2016 and YC2017). A total of 4,851 individuals from 181 families were tested, and a subset (n∼1,535) belonging to 122 families was genotyped using a ∼57K Affymetrix Axiom array. The survival against the RGNNV showed low to moderate heritability with observed scale estimates of 0.18 and 0.25 obtained using pedigree vs. genomic information, respectively. The genome-wide association analysis showed a strong signal of quantitative trait loci (QTL) at LG12 which explained ∼33% of the genetic variance. The QTL region contained multiple genes (*ITPK1*, *PLK4*, *HSPA4L*, *REEP1*, *CHMP2*, *MRPL35*, *and SCUBE*) with *HSPA4L* and/or *REEP1* genes being highly relevant with a likely effect on host response in managing disease-associated symptoms. The results on the accuracy of predicting breeding values presented 20–43% advantage in accuracy using genomic over pedigree-based information which varied across model types and applied validation schemes.

## Introduction

The aquaculture industry is expected to fulfill an increased demand of fish for human consumption as fisheries realize their resources reach their limits. The need to increase production and fulfill current and future demands comes together with challenges in long-term sustainability and production value, such as diseases outbreaks, feed supplies, water pollution, and global warming.

Diseases are a major challenge affecting fish welfare and financial losses for the aquaculture industry, particularly those from viral nature due to high mortalities and reduced growth on affected fish. Viral nervous necrosis (VNN)—also known as viral encephalopathy and retinopathy (VER)—is one of the main infectious diseases affecting marine aquaculture. The disease is caused by the red-spotted grouper nervous necrosis virus (RGNNV) which belongs to the genus *Betanodavirus* of the family Nodaviridae (OIE, 2019). VNN has been found in up to 120 species from marine and freshwater environments including European sea bass (*Dicentrarchus labrax*), the most cultured species in the Mediterranean whose annual production reached 168,642.57 tonnes in 2018 valued on $1,03 billion USD, FAO. Recently, a reassortant betanodavirus (RGNNV/SJNNV) has also been reported in gilthead sea bream (*Sparus aurata*) with a postulated asymptomatic contagious host for transmission of disease to European sea bass (Toffan et al., 2017).

Viral nervous necrosis can be transmitted horizontally and vertically (Hazreen-Nita et al., 2019). The acute phase of the disease has been associated to the elevated sea temperature where nervous signs appear, while the sub-acute form is described as necrosis on the upper jaw and head regions and has been linked to lower temperatures (Le Breton et al., 1997). VNN is cataloged as the most important problem in the Mediterranean mariculture (Vendramin et al., 2016), leading a number of attempts to produce a vaccine (e.g., Stein et al., 2013; Valero et al., 2016; Gonzalez-Silvera et al., 2019). However, despite the approval of a commercial vaccine to protect sea bass against the most common VNN genotype in the Mediterranean, its use on mass scale is still limited due to its cost as single vaccine and technical and logistic problems presented when combined with other vaccines against vibriosis and pasteurellosis. In addition, the global warming trend and the temperature dependent nature of VNN remain a threat for the sustainable production of this species (Costa and Thompson, 2016).

Selective breeding is an effective tool to continuously improve traits such as disease resistance (Gjedrem and Rye, 2018), usually by evaluating the trait on sibs of breeding candidates. Reduction in the genotyping cost and turnaround time for a large number of markers per sample has opened the door to move and implement individual selection in sib-evaluated traits such as disease resistance; this is performed either by using markers linked to a major gene influencing the trait or by implementing genomic selection (GS), leading to higher genetic gains compared to more traditional methods (Dekkers, 2007). Such programs have become a routine to improve economically important traits such as survival against pathogens in species such as Atlantic salmon, rainbow trout, and Nile tilapia.

Major factors influencing the success of a breeding program are high quality, robust and reproducible phenotypic data, and the maintenance of a reliable pedigree; furthermore, when introducing advanced genomic methods, this extends to the use of genome-wide distributed, reliable, reproducible molecular genetic markers (e.g., single nucleotide polymorphisms, SNPs). The GS provides the opportunity to rank individuals within and across families with higher accuracy (Meuwissen et al., 2001). The availability of resources including reference genomes, linkage maps, genotyping arrays, etc., convenes the applicability of the GS along with genome-wide association studies. Not long ago, the reference genome of European sea bass became available (Tine et al., 2014), and very recently, the SNP genotyping Axiom arrays were also developed, which are a part of the public domain now (Griot et al., 2021; Peñaloza et al., 2021). There are several studies on different aquaculture species where quantitative trait loci (QTL) were detected for traits of economic and welfare importance using medium- to high-density SNP arrays. The major QTLs detected in Atlantic salmon for different traits include resistance against the infectious pancreatic necrosis virus (Houston et al., 2008; Moen et al., 2009), resistance against the piscine myocarditis virus (Boison et al., 2019; Hillestad and Moghadam, 2019), age at sexual maturity (Barson et al., 2015), and fillet color (Baranski et al., 2010). QTLs have also been detected in European sea bass with moderate to low impact. The main targeted traits in sea bass for QTL detections included body weight, resistance against VNN disease, and stress resistance (Chatziplis et al., 2007; Palaiokostas et al., 2018; Chatziplis et al., 2020; Griot et al., 2021). The detected QTLs with major effects can assist in implementation of cost-effective marker-assisted selection (MAS). However, among other factors, the nature of trait(s) in a breeding program affected by a few vs. many genes along with the strategy of selection/culling within the course of the growth cycle plays a major role for the adoption/application of advanced selection methods (MAS vs. GS). The GS has shown the advantage over the classical pedigree method by enabling the prediction of breeding values for the candidates with higher accuracy (Tsai et al., 2016; Correa et al., 2017; Vallejo et al., 2017; Aslam M. L. et al., 2020; Aslam M. L. et al., 2020). The application of the GS becomes highly important and efficient for lowly heritable and difficult traits which are usually not recorded on live candidates (e.g., carcass quality and disease resistance traits which are currently recorded on sibs of live selection candidates) and also allows selecting the best individuals for such traits instead of best families with traditional methods.

In this study, we investigated the genetic variation for resistance to the RGNNV from a challenge test in a commercial European sea bass breeding program performed during two consecutive years. In addition, fish from the same challenge tests were genotyped to detect QTL for survival against the RGNNV and assess the potential of the GS to improve resistance against VNN.

## Materials and Methods

### Experimental Population

The presented results are based on challenge testing of two different year classes in the Nireus SA’s breeding nucleus of European sea bass (Thorland et al., 2017; Nirea et al., 2021). The commercial family-based breeding program operates with overlapping generations, and the presented experimental population is part of year classes 2016 (YC2016) and 2017 (YC2017). These year classes have a selection history of between three and four full cycles of selection from families in base population (year-classes 2004 and 2005) and are in the program defined in generations between F3 and F4. Families in the breeding program are produced by artificial stripping of mature females and males and subsequent controlled crossings in the family design including both maternal and paternal half-sibs.

Parents to both year classes included in this study were photoperiod-manipulated to mature out of the natural spawning season. YC2016, consisting of 89 full-sibs families produced from 65 sires and 30 dams, generated between September 3 and October 10, 2016; YC2017 consisted of 92 full-sib families derived from 85 sires and 35 dams produced by artificial stripping in the period September 17 to October 13, 2017. In the mating design, each female parent was mated with one to four males with an average of 2.8 for YC2016 and 2.6 for YC2017. Each male parent was mated with one to four females (on average 1.4 females per male) in YC2016, whereas in YC2017 each male parent was mated to one to two with an average of 1.1 females per male. The average age of the male broodstock was 3.9 years post-hatching for YC2016 and 3.6 years for YC2017, while for the female broodstock, the average age was 3.5 and 4.0 years for YC2016 and YC2017, respectively. The use of broodstock of different years of age and the structure with overlapping generations builds a strong genetic tie between year classes in the breeding nucleus which allows for combined analysis of data from both year classes in this experimental population.

Families were reared in separate circular tanks of 315-L capacity at the Enalios-Breeding Programme hatchery of Nireus, S.A., located in Central Evia. Approximately 30 individuals per family were individually PIT-tagged using 8-mm Biomark tags. The PIT tagging for YC2016 was performed at an average body weight of 10.9 g (sd = 2.5 g) between March 21 and April 12, 2017, whereas the PIT tagging for YC2017 was performed from March 20 to March 23, 2018, at an average weight of 8.9 g (sd = 1.7 g). All PIT-tagged individuals within each year class for the specific challenge test were stocked in a single tank after transportation to the challenge test facilities at the laboratory of Ichthyology, Aquaculture and Diseases of Aquatic Organisms (ICHTHYAI), Department of Marine Sciences, at the University of Aegean in Lesvos, Greece. The date of transportation of individuals to the challenge test facility was April 24 in 2017 (YC2016) and June 13 and 15 in 2018 (YC2017).

### Challenge Tests for Viral Nervous Necrosis

Both year classes (YC2016 and YC2017) were challenged according to similar protocols using a European sea bass RGNNV isolate from a commercial fish farm outbreak in 2012, and the virus challenge was performed with intramuscular (IM) injection. In YC2016, sea bass individuals with an average weight of 14.4 g were injected with a challenge dose of 10^6^ TCID_50_/ml, defined from in-house pre-trials. The fish were injected on May 13, 2017 and distributed randomly to three experimental tanks of 2 m^3^, and mortalities were recorded every 4 h for a period of 28 days after infection (with no exceptions including recordings during nights and weekends), until mortality had stopped. The second challenge test was conducted on YC2017 (June 24, 2018) with similar conditions and environment as on YC2016, but the infection dose was 5 × 10^6^ TCID_50_/ml. YC2017 had an initial average body weight of 25.3 g in the test. All fish were fin-clipped before the challenge test, and tissues were preserved in 95% ethanol and stored at −4°C.

### Genotyping

In order to reduce genotyping costs, challenge-tested individuals (*n* = 4,851) were subsided from both year classes (YC2016 and YC2017). Hence, out of total 4,851 individuals, a subsample of 1,535 individuals were selected for genotyping comprising 767 individuals belonging to 30 families of YC2016 and 768 individuals from 92 families of YC2017. Since the accuracy of selection using genomic information would be highly dependent on the relationship of training (the training group is referred to the individuals with phenotype information available from the same generation, normally the full sibs of candidates) and tested (referred to individuals without phenotypic records i.e., the candidate group) population, pedigree relationships were considered to select individuals from families with genetic links to contemporary families. Tissue samples from selected fish were used for the DNA extraction and genotyping using the SNPs-based ∼57K Affymetrix Axiom array (DlabCHIP, Griot et al., 2021), and the position of markers was determined based on the genome build 1.0 of European sea bass (seabass_V1.0, Tine et al., 2014). Genotyping was performed at the Gentyane facility, Clermont-Ferrand, France.

The raw genotype data were quality-filtered by PLINK software (Purcell et al., 2007). SNP markers with the minor allele frequency (MAF) lower than 5%, missing rate higher than 10%, and those not passing the Hardy–Weinberg equilibrium exact test (*p* < 1.0 × 10^−6^) were excluded from the data. Filtering was also performed at the individual level with individuals removed based on the missing genotype rate (>10%) and poor heterozygosity (higher than ±3sd of population). The filtering step retained a total of 1,489 individuals (754 and 735 for YC2016 and YC2017, respectively) from 120 full-sib families of which 28 families represented YC2016 (15–30 sibs per family), and 92 families from YC2017 (4–14 sibs per family) genotyped with approximately 52K SNPs.

### Statistical Analyses

**Data description and statistics:** The statistics for the recorded traits and the initial evaluation of models were obtained using the generalized linear model in statistical software “R”. With the full dataset (*n* = 4,851) year classes and the family effects were tested; year classes did not show significant effects (*p* = 0.106), while the family effect was highly significant with *p* < 0.001. Though the effect of year classes was not significant, it was still used in the model to avoid year class-specific deviations in estimates. The analysis and/or results presented below are mainly focused on using the combined dataset from both year classes (YC2016 and YC2017) unless otherwise specified.

**Quantitative genetic parameter estimation:** Estimation of genetic parameters was conducted for the binary survival trait recorded at the end of test(s) (1 = survival; 0 = dead). The analysis was conducted including either all data (n_ind_ = 4,851) or the genotyped subset of the data (n_ind_ = 1,489) summarized in Table 1. Two models (Model-1 and Model-2) were applied to estimate the variance component by ASReml software (Gilmour et al., 2015). Model-1 was the linear mixed animal model which can be written as follows:

y=Xb+Ta+Zc+e, (1)

where y is the observed survival status in the challenge test; b is a vector containing the overall mean and fixed effect of year class; a is a vector of additive genetic effects with a distribution $∼N\left(0,\left[A/G\right]\sigma_{a}^{2}\right)$ , where A is the numerator relationship matrix calculated from the pedigree (for pedigree-based estimates); G is a genomic relationship matrix (for genomic estimates) computed using VanRaden (Vanraden, 2008); c is a vector of random effects common to full sibs caused by other factors than additive genetics (i.e., including environmental tank effects caused by the separate rearing of full-sib families until individual tagging, non-additive genetic effects, and maternal effects) with distribution $∼N\left(0,I\sigma_{c}^{2}\right)$ ; and e is the vector of random environmental effects with distribution $∼N\left(0,I\sigma_{e}^{2}\right)$ **.** X , T , and Z are the assigned design matrices to the respective vectors b , a , and c .

**TABLE 1:** Data statistics for the survival trait.

	Phenotyped				Genotyped			
Population	N Fam	N fish	Fish/family	%Survival	N Fam	N fish	Fish/family	% Survival
YC2016	89	2499	28.1	42	30	767	27.4	46
YC2017	92	2352	25.7	41	92	768	8.4	44
Total/Mean	181	4851	26.9	42	122	1535	17.9	45

Model-2 was a sire-dam model where the binary data (1 = survival; 0 = dead) were fitted to a threshold model using the “probit” link function. In matrix notation, the model can be written as follows:

$$y=\mathrm{Xb}+\left(T_{s}+T_{d}\right)u+\mathrm{Zc}+\mathrm{e,}$$ (2)

where  y **,** Xb **,** Zc , and e components of the model are the same, as explained earlier; u is a vector of half the sire-dam additive genetic effects with a distribution $∼N\left(0,A¼\sigma_{a}^{2}\right)$ , where sire and dam were constrained to have equal additive genetic variance ( $\sigma_{s}^{2}=\sigma_{d}^{2}=\sigma_{u}^{2}=¼1/4\sigma_{a}^{2}$ ); the component A in distribution refers to the relationship matrix among the parents (sire and dams); $T_{s}$ and $T_{d}$ are the assigned design matrices linking sire and dam to the respective value of u .

From Model-1, heritability was estimated as h2=σa2σp2 , whereas from Model-2, it was estimated as h2=4σs2σp2 . For both models, the non-additive effect common to full sibs was estimated as c2=σc2σp2 **,**

where $\sigma_{a}^{2}$ is the additive genetic variance; $\sigma_{s}^{2}$ is the additive genetic sire variance; $\sigma_{c}^{2}$ is the non-additive variance common to full sibs; $\sigma_{e}^{2}$ is the residual variance; and $\sigma_{p}^{2}=\sigma_{a}^{2}+\sigma_{c}^{2}+\sigma_{e}^{2}$ for results from model-1 and $\sigma_{p}^{2}=2\sigma_{s}^{2}+\sigma_{c}^{2}+\sigma_{e}^{2}$ for results from model-2.

**Genome-wide association analysis (GWAS):** Genome-wide association analysis was performed using the linear mixed animal model implemented in the GCTA program with the “--mlma-loco” function (Yang et al., 2011a). The “--mlma-loco” function allows the estimation of an SNP effect by accounting the additive genetic variance expressed by all the markers distributed over all the linkage groups other than the linkage group which contains the SNP. The method of leaving one chromosome out (the chromosome carrying the marker in question for which the effect and association must be computed) increases the power of the association analysis. This removal of the marker in question and all the other linked markers from the chromosome avoids double-fitting/adjusting in the model, both as a fixed effect tested for association and as a random component by including in the genomic relationship matrix (Yang et al., 2014). The model used in GWAS was similar to Model-1 except that the first five eigenvectors computed from the genomic relationship matrix were included as covariates in the model, and the common environment effect was excluded. The eigenvectors as covariates in genome-wide association analysis are usually used to adjust for population stratification and control spurious genetic associations caused by false linkages of markers with the population structure instead of true marker trait associations (Price et al., 2006). The G **-**matrix was computed according to the VanRaden (Vanraden, 2008) method as $\frac{PP^{'}}{2*\sum_{i=1}^{Nsnp}p_{i}\left(1-p_{i}\right)},$ where P , Nsnp , and $p_{i}$ are the matrix of centralized genotypes, total number of SNP markers, and the allele frequency of the reference allele, respectively.

The SNP markers were categorized as genome-wide significant when they surpass the Bonferroni threshold for multiple testing *p*-value of $P\leq 2.37\;\times 10{\;}^{-07}$ with $-log_{10}\left(P\right)=6.62$ , or if they surpassed the *p*-value of $P\leq 5.69\;\times 10{\;}^{-05}$ with $-log_{10}\left(P\right)=5.24$ , then they were classified as suggestive if they surpassed the *p*-value P≤0.05 . The significant values (chromosome and/or genome-wide) were computed, as described in the study by Aslam M. L. et al. (2020). The observed $-log_{10}\left(P-values\right)$ for all the SNPs were plotted against expected $-log_{10}\left(P-values\right)$ from a theoretical distribution. The inflation factor (lambda, λ) was calculated using $\text{λ}=\frac{median\left(\chi^{2}\right)}{0.456}$ to assess deviations (inflation/deflation) in *p*-values.

**Estimation of the SNP(s) variance:** The proportion of the genetic variance explained by the top significant SNP(s) were computed using two methods. One is the direct method where genetic variances of SNPs were computed using allele frequencies and allele substitution effects as $2p_{i}q_{i\alpha_{i}^{2}}$ (Hill and Mackay, 2004). The other is the indirect method where the highest significant SNP(s) from the GWAS analysis were used as additional fixed effect(s), as explained in the study by Aslam et al. (2018). The statistical model used for the indirect method was the same as described under GWAS, but the G matrix was constructed with all other SNPs except the SNP(s) used as a fixed effect. The proportional reduction in the total genetic variance due to the addition of the fixed effect of the SNP(s) was considered as the contribution from the SNP(s).

**Breeding value estimation:** Breeding values for the survival (binary, 0/1 trait) against VNN were computed through model-1 without using random effects common to full-sibs ( c ). The estimates of breeding values were obtained using a customized script involving R/BGLR (Pérez and De los Campos, 2014) and the ASReml (Gilmour et al., 2015) programs. The obtained breeding values were used to evaluate the accuracy of predictions acquired through models involving information from pedigree (PBLUP), genomic, i.e., GBLUP, BayesB, BayesC, and Bayesian Lasso (Park and Casella, 2008; Habier et al., 2011), and the hybrid information matrix, i.e., HBLUP, also known as single-step genomic evaluation, where the relationship matrix could link genotyped and ungenotyped individuals (Legarra et al., 2009).

**Accuracy of prediction:** For the evaluation of accuracy of predictions, two datasets were used, 1) genotyped individuals (*n* = 1,489) and 2) a full dataset (*n* = 4,851, genotyped and ungenotyped individuals). The comparison for the accuracy of predictions among the genomic and pedigree models was performed using genotyped individuals only, while PBLUP and HBLUP models were compared using the full dataset (*n* = 4,851).

To compare prediction accuracies, validation schemes were designed in two ways.

1) Within family masking: Under this scheme, 30% of individuals within each family were randomly masked, which produced 447 individuals as validation animals, and the remaining 1,042 individuals with available phenotypes were kept as the training set.
2) Random masking: Under this scheme, 30% of the individuals were randomly masked without giving any consideration on families. Hence, 1,042 individuals were used for training and 447 as validation animals.

The adopted schemes for the comparison of PBLUP vs. HBLUP were the same as 1) and 2), but the number for validation and training individuals ended up large with 1,455 validation and 3,396 training animals. The breeding values for the masked individuals were computed using pedigree, genomic, and the hybrid relationship matrices The mean accuracy of 20 replicates was computed as the correlation ( $r_{\mathrm{corr}})$ of the estimated breeding values (pedigree/genomic/hybrid) with the pre-adjusted phenotype, $y_{\mathrm{adj}}$ , which was scaled by the square root of the heritability as $r_{\mathrm{corr}}=\frac{\rho \left(P\left[G|H\right]\mathrm{EBV},y_{\mathrm{adj}}\right)}{\surd h^{2}}$ , where ρ , P[G|H]EBV , and $h^{2}$ are correlation coefficients, breeding values estimated using pedigree or genomic or hybrid information, and pedigree-based heritability estimates ( $h^{2}$ **=** 0.18), respectively.

## Results

### Challenge Test

**FIGURE 1:**
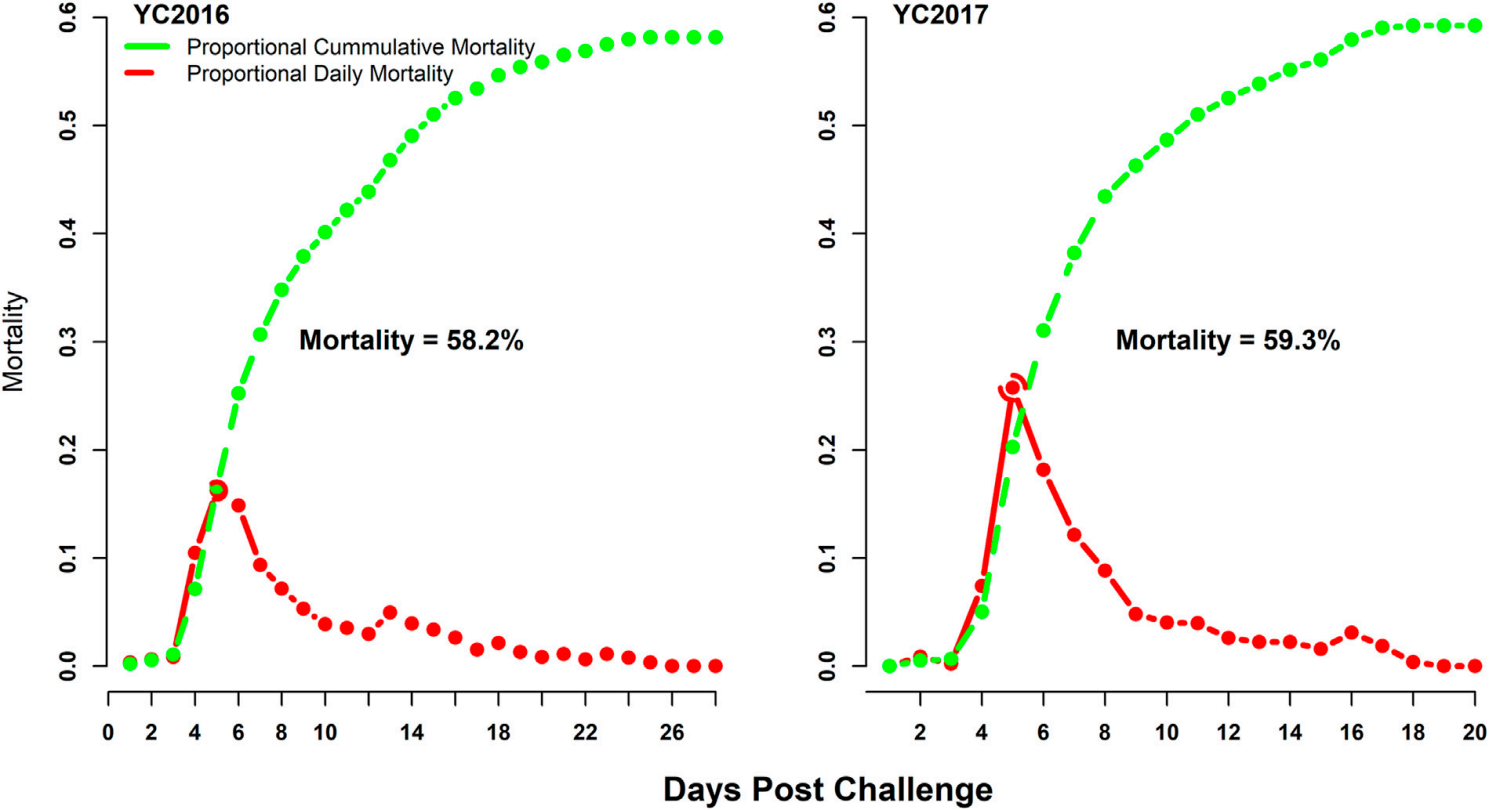
Distribution of mortalities in YC2016 and YC2017 during the challenge test against the nervous necrosis virus.

The challenge test resulted significant and very similar mortalities with 58.2 and 59.3% for YC2016 (Bakopoulos et al., 2018) and YC2017, respectively (Figure 1). Mortalities started to appear during the first and second day after infection in YC2016 and YC2017, respectively, reaching a peak during day 5 for both challenge tests. Mortalities ceased before termination of the trial approx. on day 20 after challenge. All mortalities were recorded, and the individuals who survived until day ∼20 from the start of the challenge were considered as survivors (alive). The distribution of mortalities in each full-sib family along with the distribution of full-sibs per family is given in Supplementary Figure S1.1.

### Data Statistics

The recorded data contained 4,851 observations ( $n_{YC2016}$ = 2,499 and $n_{YC2017}$ = 2,352) from pedigreed individuals while the subset of these pedigree individuals 1,489 also had genotype information with ∼52K SNPs. The average survival in the large dataset (*n* = 4,851) and the subset (*n* = 1,489) of the data was very similar, with 42 and 45%, respectively (Table 1).

### Genetic Parameters

The observed and the liability scale heritability estimates obtained using genomic information were higher than the pedigree-based estimates (Table 2). The observed scale heritability estimates obtained using the large dataset (including YC2016 and YC2017) were 0.18 ± 0.03 and 0.25 ± 0.04 for pedigree and genomic information, respectively. The liability scale heritability estimates obtained using pedigree information were 0.27 ± 0.04 while the same scale estimates with genomic information were 0.40 ± 0.06, respectively. The estimates for the environment common to full sibs, $c^{2}$ were close to zero and non-significant with either kinds of model or source of information. The highest obtained estimates of $c^{2}$ were 0.02 ± 0.03 through the sire-dam threshold model used on YC2017 data.

**TABLE 2:** Genetic parameters with standard errors for survival at the end of the VNN challenge test.

	Pedigree estimates				Genomic estimates		
Dataset/Models	LM		$TM_{\mathrm{sire-dam}}$		LM		TM
Components	$h^{2}$	$c^{2}$	$h^{2}$	$c^{2}$	$h^{2}$	$c^{2}$	$h^{2}$
Year 2016	0.15 ± 0.04	0.00 ± 0.00	0.23 ± 0.05	0.00 ± 0.00	0.26 ± 0.04	0.00 ± 0.03	0.40 ± 0.10
Year 2017	0.15 ± 0.06	0.02 ± 0.02	0.23 ± 0.09	0.02 ± 0.03	0.20 ± 0.06	0.00 ± 0.02	0.32 ± 0.10
Year 2016 + 2017	0.18 ± 0.03	0.00 ± 0.00	0.27 ± 0.04	0.00 ± 0.00	0.25 ± 0.04	0.00 ± 0.02	0.40 ± 0.06

LM, linear animal model; TM_sire-dam_, sire-dam threshold model; TM, estimates computed using the conversion equation (Lee et al., 2011) from the observed scale to the liability scale; h^2^, heritability; c^2^, random effects common to full sibs.

### Genome-wide Association Analysis

**FIGURE 2:**
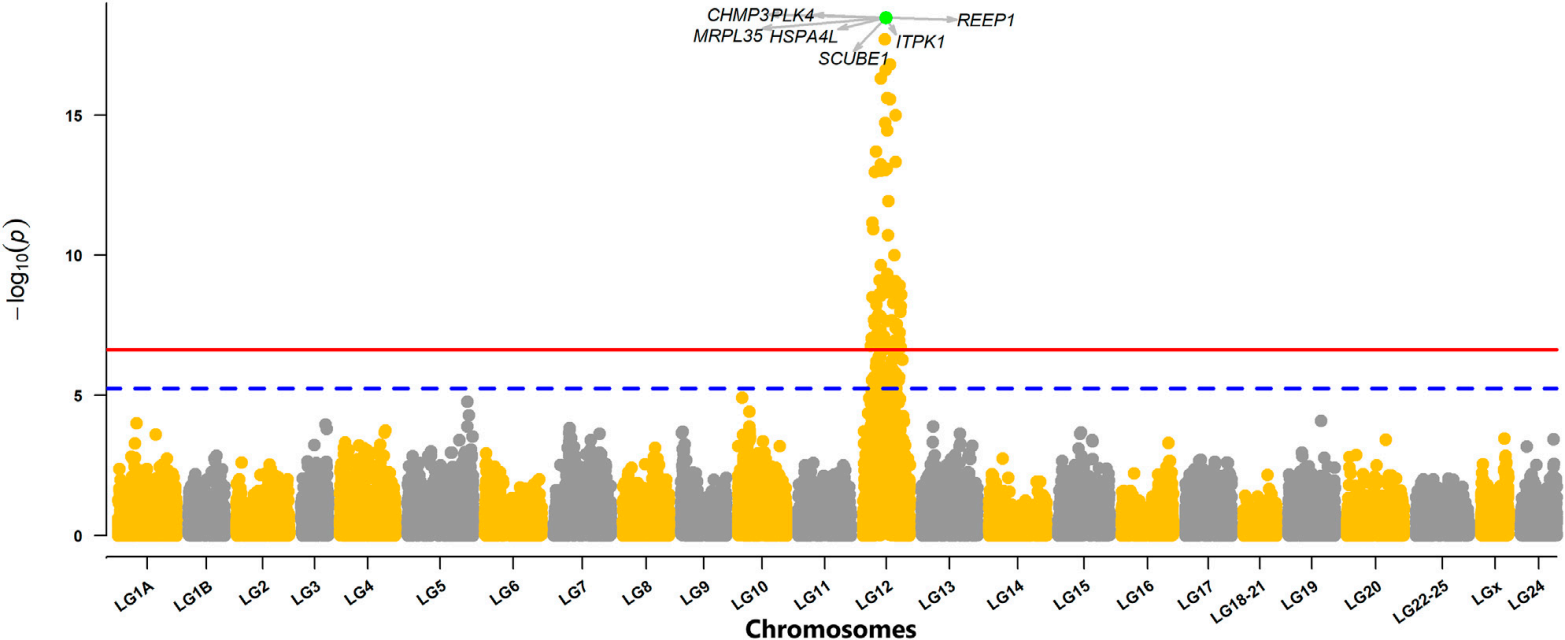
Manhattan plot with the distribution of −log10(p) values of SNPs across different chromosomes. The solid red line represents the genome-wide Bonferroni-corrected threshold, while the dashed blue line is the chromosome-wide Bonferroni-corrected threshold. The green highlighted point is the highest significant SNP of GWAS analysis, and the arrows are depicting underlying genes within the ±25 Kb region from the position of the highest significant SNP.

**FIGURE 3:**
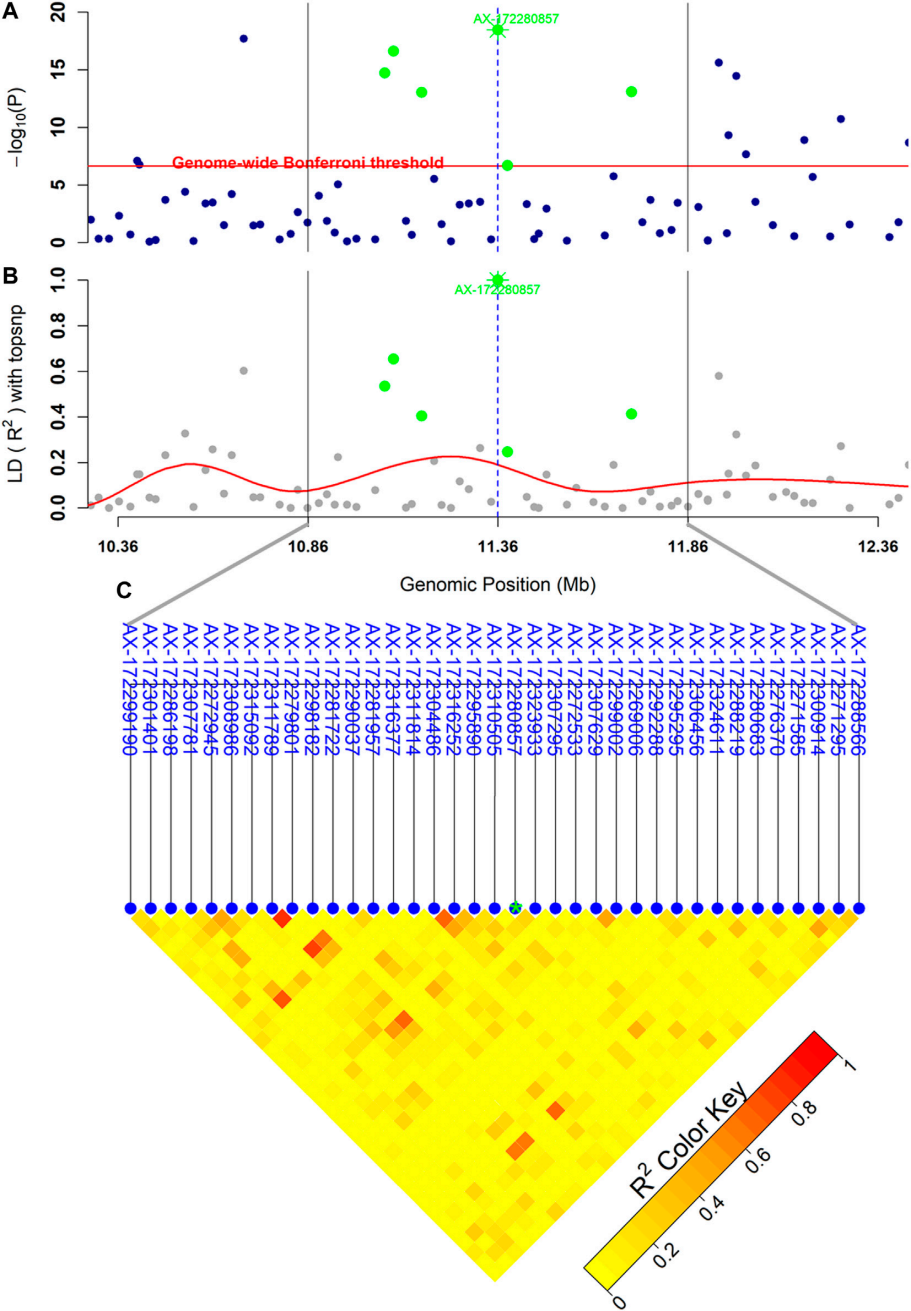
High-resolution depiction of the QTL region at LG12 with linkage disequilibrium information. **(A)** displays the distribution of −log10(p) in the QTL region, the horizontal red line represents the genome-wide Bonferroni-corrected threshold, **(B)** exhibits the LD of SNPs with the highest significant SNP, the solid red line shows the pattern of LD decay from the highest significant SNP, and **(C)** shows the LD among the SNPs within 1.0 Mb (10.86–11.86 Mb). The green dots denote the genome-wide significant SNPs, while the asterisk dot is the highest significant SNP of GWAS analysis. The dashed vertical blue line shows the position of the highest significant SNP, and the fainted vertical gray lines mark the 1.0 Mb region with ±500 Kb from the highest significant SNP.

The GWAS analysis from the combined dataset of both year classes (YC2016 and YC 2017) revealed a strong signal of QTL at LG12 with 72 SNPs crossing the genome-wide threshold (Figure 2). Moreover, the analyses on individual year class (YC2016 or YC2017) were also performed which showed a consistent strong peak of QTL at LG12 (Supplementary Figure S1.2). The details for the top ten highest significant SNPs with their position in genome and allele substitution effects, along with the genetic variances explained by the SNPs, are given in Table 3. The detected genome-wide significant SNPs (*n* = 72) cover a large region of 15 Mb (3,778,017 bp to 18,822,298 bp) with the top significant SNP AX-172280857 centered at 11,359,282 bp (Figure 3). However, when the most significant SNP (AX-172280857) was used as an additional fixed effect in the model (as explained under the indirect method for computation of SNP variance), none of the SNPs showed any chromosome and/or genome-wide significance, indicating that there is only one QTL in the region (Supplementary Figure S1.3). A quantile–quantile plot (QQ-plot) obtained using $-log_{10}\left(P-values\right)$ acquired from the genome-wide association analysis is presented in Supplementary Figure S1.4. The obtained genomic inflation factor (lambda value, λ) with the applied GWAS model was 1.168. The inflation factor of 1.168 seems to suggest slight inflation of *p*-values even with the application of first five eigenvectors as covariates in the model to possibly correct the existed population structure (Supplementary Figure S1.5). However, much larger lambda values can also be obtained when GWAS is performed with a large sample size using high-density genome-wide distributed markers (Yang et al., 2011b).

**TABLE 3:** The top 10 significant SNPs detected in GWAS analysis ranked with respect to the level of significance.

SNP-ID	LG	Pos(bp)	A1	A2	MAF	α	SE	*p*	$\mathrm{\%Va}r_{G}$
AX-172280857	12	11359282	C	T	0.115	0.289	0.032	3.42E-19	26.204
AX-172273041	12	10690280	C	T	0.103	0.314	0.036	2.02E-18	27.120
AX-172298845	12	13410063	A	C	0.078	0.325	0.038	1.60E-17	22.638
AX-172279801	12	11085391	A	G	0.078	0.323	0.038	2.52E-17	24.046
AX-172305328	12	8782984	G	A	0.265	0.203	0.024	4.95E-17	24.250
AX-172296534	12	11941161	C	A	0.165	0.230	0.028	2.49E-16	22.636
AX-172277566	12	13335186	G	A	0.104	0.277	0.034	2.74E-16	21.804
AX-172278329	12	16168988	C	A	0.108	0.287	0.036	1.02E-15	24.165
AX-172311789	12	11061935	A	G	0.087	0.312	0.039	1.90E-15	26.025
AX-172310909	12	11986782	G	A	0.172	0.216	0.027	3.52E-15	20.241

The SNPs are sorted based on their level of significance with LG, linkage group; Pos(bp), physical position of SNP; A1 & A2, Minor and major alleles, respectively; MAF, minor allele frequency; *α*, allele substitution effect for A1 allele; SE, standard error; *p*, significance value; $\mathrm{\%Va}r_{G}$ , proportion of the genetic variance explained using the direct method. The SNP positions are based on genome build 1.0 (seabass_V1.0, Tine et al., 2014) of European sea bass.

**SNP variance:** The proportion of genetic variance explained by the highest significant SNP using the direct method was ∼26.0% (Table 3). The application of the highest significant SNP as an additional fixed effect in the GWAS model caused reduction of genetic variance from 0.06 to 0.04 (Supplementary Table S1.1). Hence, the proportion of genetic variance explained by the highest significant SNP using the indirect method was 33.33%, which is a proportional reduction in the genetic variance with the application of the highest significant SNP as an additional fixed effect.

### Accuracy of Predictions

**FIGURE 4:**
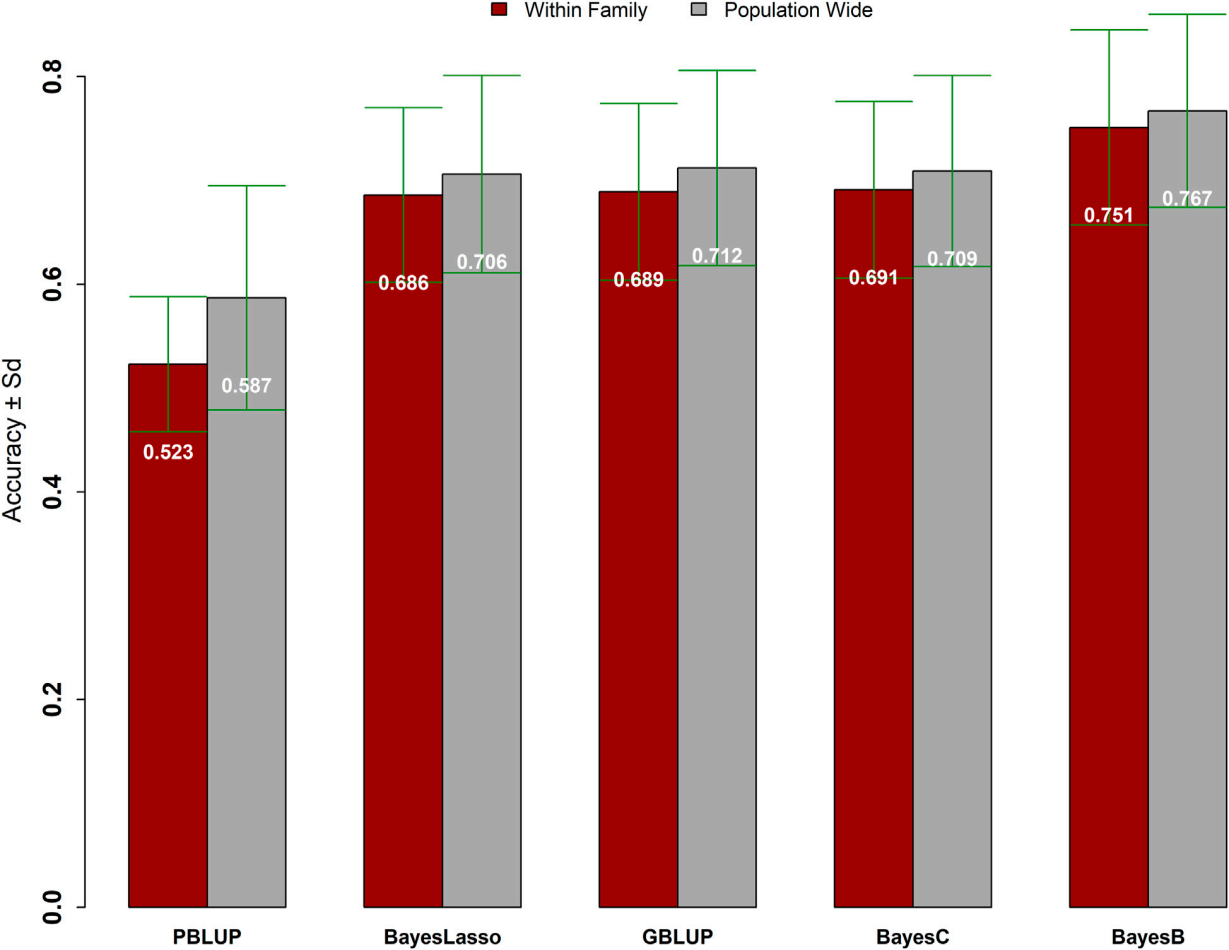
The accuracies of prediction for resistance against VNN using pedigree vs. genomic models.

Accuracies of prediction using pedigree vs. genomic information are plotted in Figure 4. Regardless of validation schemes applied, the mean accuracies obtained using genomic information were significantly higher than the pedigree information-based accuracies (Figure 4 and Supplementary Table S1.2). The mean accuracy achieved from the genomic models (GBLUP, BayesB, BayesC, and Bayesian Lasso) was 0.71, while the mean accuracy from the pedigree-based PBLUP model was 0.55 (Figure 4 and Supplementary Table S1.2). The mean accuracy of 0.70 and 0.72 was achieved with the genomic models using validation scheme **“a”** (within family masking) and validation scheme **“b”** (random masking), respectively. A similar trend was also seen using the PBLUP model with higher mean accuracy obtained with validation scheme **“b”** ( $r_{corr}$ = 0.59) compared to **“a”** ( $r_{corr}$ = 0.52).

The comparison among the genomic models with respect to the prediction accuracy for survival against VNN showed that the Bayesian models worked either better or equally well as GBLUP. The trend in the rank on performance of genomic models was the same within both validation schemes (“a” and “b”). The BayesB model displayed the highest accuracy with estimates of 0.77 ± 0.09 and 0.75 ± 0.09 for validation schemes **“b”** and **“a”,** respectively. The other genomic models (BayesC, Bayesian Lasso, and GBLUP) showed approximately similar accuracy of prediction with fractional differences with values 0.69 ± 0.09 and 0.71 ± 0.09 for validation schemes **“a”** and **“b”**, respectively (Figure 4 and Supplementary Table S1.2).

The comparison of the prediction accuracy for PBLUP vs. HBLUP models showed higher accuracies obtained with the HBLUP model, with estimates of 0.57 ± 0.05 and 0.58 ± 0.05 for validation schemes **“a”** and **“b”,** respectively (Supplementary Figure S1.6). The prediction accuracy for the PBLUP model was 0.52 ± 0.05 and 0.53 ± 0.05 with validation schemes **“a”** and **“b”,** respectively.

## Discussion

Viral nervous necrosis is one of the highly contagious and deadliest diseases for many aquaculture species which also pose a serious threat for European sea bass producers, with fry and juveniles being highly susceptible. The outbreak of VNN may cause up to 100% mortalities at larval and around 20% mortalities at advanced juvenile stages (Le Breton et al., 1997; Munday et al., 2002). Moreover, the surviving fish present poor growth rate and ultimately high economic losses for the producers. In the current study, we explored the genetic basis of resistance against the RGNNV using large-scale challenge experimentation, phenotype recording, and high-density genotyping using SNP markers with ultimate intentions to improve fish health and welfare, leading to sustainable production.

The challenge test results from the current study presented steady/predictable survival/mortality curves, with a peak in mortality at day 5 after infection and sharp reduction of mortalities at day 11 after infection, which were similar to results reported by Doan et al. (2017) and Faggion et al. (2021) where individuals were infected *via* intraperitoneal and intramuscular injections, respectively. The reported trend from the studies of Doan et al., and Faggion et al., was followed as peak mortalities at days 6 and 4 after the infection and the sharp decrease in mortalities from days 7 and 9 after infection, respectively. Contrarily, Palaiokostas et al. (2018) reported smoother mortality curves with mortality peaks at days 8 and 15 post-infection where fish were infected by the immersion model. In another study conducted by Griot et al. (2021), the immersion-based challenge test that was performed showed variable mortality/survival curves (specific to each population) with survival ranging from 38 to 79% and peak mortalities at day 10 post-infection. Hence, the behavior of the mortality curve may slightly deviate depending on experimental population and the type of the infection model used. The level of peak mortalities reaches in the first week when applying injection-based infection models and may move to the second week of infection with immersion models. The shift/delay in reaching the peak mortality from day 4 or 5 [(Faggion et al., 2021); current study] to day 10 or 15 (Palaiokostas et al., 2018; Griot et al., 2021) and the duration of challenge tests (∼40–49 (Palaiokostas et al., 2018; Griot et al., 2021) vs. 20–28 days (Faggion et al., 2021); current study) meet the expectations because intramuscular injection-based models skip mucus and skin barriers which are also a part of the defense system in case of immersion-based models that will ultimately delay the time to infection and immune response of the host.

The current study revealed moderate heritability estimates for survival to VNN (Table 2) which are concordant with those reported in the literature (Palaiokostas et al., 2018; Faggion et al., 2021; Griot et al., 2021). However, a few extreme estimates of heritability as high as 0.59 (observed scale) and 0.84 (on underlying liability scale) were also reported by Griot et al. (2021) which were computed using data recorded on four backcross families. In general, the estimates of heritability for survival against VNN are quite consistent and robust probably because the history of breeding for European sea bass is not very long, and possibly, selection-based divergence has not taken place for this trait in farmed populations.

The GWAS analysis of the current study presented a strong signal of QTL at LG12 which was very consistent across datasets coming from two different year classes, though both year classes YC2016 and YC2017 were exposed to the virus in two independent challenge tests (Supplementary Figure S1.2). Moreover, the study from Griot et al. (2021) also reported very stable QTL detected at the same region of LG12 using composite interval mapping analysis. However, Griot et al. (2021) also detected multiple other putative QTLs at LG8, LG15, and LG19 with weaker signal crossing the chromosome-wide significance threshold. The putative QTLs of LG15 and LG19 in the study by Griot et al. (2021) were also consistent with our results when GWAS analysis was performed on YC2016 only (Supplementary Figure S1.2). The inconsistency of QTLs detected at LG15 and LG19, especially with the increased power by the added dataset from YC2017 perhaps indicates false signals possibly due to pseudo-linkages among markers which turn out to be insignificant with added datasets from relatively less-related individuals of YC2017. A study from Palaiokostas et al. (2018) also reported putative QTLs (signals crossing the chromosome-wide threshold) located at LG3, LG20, and the markers from unassigned scaffolds which were not detected either in our study or the study from Griot et al. (2021). However, a single QTL in the current study vs. multiple detected QTLs in both studies by Griot et al. (2021) and Palaiokostas et al. (2018) might be due to the genetic differences in populations and/or may reflect the differences in challenge tests (immersion vs. injection models) as infecting the fish by the immersion method will involve both specific and non-specific immune responses (through skin and mucus barriers) while infection through injection (this study) will skip the skin and mucus barriers and triggers specific immune responses.

The statistics of the top ten highest significant SNPs including minor allele frequencies, position in the genome, and allele substitution effects are detailed in Table 3. All the top 10 SNPs present low minor allele frequencies ranging from 0.078 to 0.265 with a similar magnitude of the allele substitution effect (ranging from 0.203 to 0.325) along with the same direction with minor alleles being favorable with the positive effect on survival (phenotype coded as 1 = survival and 0 = dead). These observations perhaps indicate that these top significant SNPs are in linkage disequilibrium (LD) and possibly are in the same phase with the causative mutation. The distribution of the survival percentage across the genotypes from the highest significant SNP (SNP-ID = AX-172280857) showed ∼118% higher survival for individuals carrying favorable genotypes (CC) than homozygous unfavorable (TT) genotypes (Supplementary Figure S1.7).

The highest significant SNP in our study explained up to 33.33% (Supplementary Table S1.1) of the genetic variance detected through the analysis of combined datasets from both year classes (YC2016 and YC2017). The proportion of the genetic variance explained by the highest significant SNP computed using the direct method (Hill and Mackay, 2004) was ∼26.20%, which is slightly lower than what is obtained using the indirect method, 33.33%. The variances computed using the direct method are largely influenced by allele frequencies and computed, considering SNPs/markers are independent genomic fragments which is normally not the case. Hence, variances of significant SNPs within the QTL region should not be summed to calculate the total genetic variance due to possible LD among the SNPs. The top ten genome-wide significant SNPs from our study showed a mean LD of 0.49 (ranging from 0.19 to 0.87, in Supplementary Figure S1.8) although the markers are distributed over a large distance of more than 7 Mb (Table 3). Moreover, the application of the highest significant SNP as a fixed effect in the indirect method causes shrinkage of *p*-values for all the other chromosomes and/or genome-wide significant SNPs at LG12 (Supplementary Figure S1.3) which further supports the argument on the existence of single QTL rather summing the variances from all the SNPs in the QTL region. The relatively large impact of single QTL (up to 33.33% of the genetic variance) does not necessarily mean that the tagged SNP (SNP-ID = AX-172280857) is a causative mutation, but this SNP explains an important part of QTL variation, either directly or through LD with the causative mutation.

The QTLs in the study from Griot et al. (2021) explained relatively small proportion (up to 9%) of total genetic variance which is approximately four times lower than what is found in the current study. These large differences might be very likely due to the differences in computational methods used (e.g., effects using Bayesian models (Griot et al., 2021) vs. effects computed using linear mixed models). The other factors which might also contribute include specific genetic variations among populations, challenge test methods (immersion vs. IM injection in our study), and/or family structures. The infection model using the immersion method is relatively less controlled which might have been influenced by the natural history of the disease such as involvement of natural external barriers of skin and mucus, random variations on pathogen loads, nonspecific immunity response, and stress among others (Oidtmann and Sitja-Bobadilla, 2017). Moreover, Griot et al. (2021) did not observe the putative QTL of LG12 when the progeny of the VNN-susceptible parent/line was challenge tested *via* IP injection, and the most of the detected QTLs were population-specific, suggesting the influence of challenge test methods and/or level of population differences. The consistency of detected QTLs of LG12 across different year classes of our study and the concordance with the results from the study by Griot et al. (2021) possibly validate the large effect of the QTL region on survival against VNN. Moreover, it also strengthens the argument that the effect of identified genomic regions at LG12 perhaps covers variation due to the specific immune-based defense mechanism of the host.

Genes underlying the QTL region i.e., the 50 Kb region covering ±25 Kb from the highest significant SNP (AX-172280857) position was searched using the European sea bass genome (seabass_V1.0, Tine et al., 2014). The QTL region contained seven genes including *ITPK1*, *PLK4*, *HSPA4L*, *REEP1*, *CHMP2*, *MRPL35*, and *SCUBE1* (Supplementary Material S1, Supplementary Table S1.3, Supplementary Figure S1.9). The upstream genes to the highest significant SNP were *ITPK1* (inositol tetrakisphosphate 1-kinase 1) and polo-like kinase 4 (*PLK4*). The *ITPK1* gene is known to be engaged with cell physiological functions involving signaling molecules inositol phosphates (Desfougères et al., 2019), and *PLK4* (polo-like kinase 4) has a role in cell replication by centriole duplication (Garvey et al., 2021). The highest significant SNP was annotated as the intronic SNP, that is, located within the intron of the *HSPA4L* (heat shock protein family A, member four like) gene which belongs to a family of heat shock proteins (*HSPs*), *HSP70* and plays a role in response to harmful circumstances and protect the cell from stress (Liu et al., 2019). Moreover, *HSPs* from the family of *HSP70* have also shown association nervous necrosis virus infection in Asian sea bass (Liu et al., 2016) and with immune system maintenance in humans (Chiricosta et al., 2020). The immediate next downstream gene after *HSPA4L* is *REEP1* (receptor expression-enhancing protein 1) which is found in nerve cells (neurons), the brain, and the spinal cord (Hurt et al., 2014). The other downstream genes from the position of the highest significant SNP were *CHMP2* (charged multivesicular body protein 2) and *MRPL35* (mitochondrial ribosomal protein L35), which are known to be involved in the physiological function in cells while *SCUBE1* (signal peptide-CUB-EGF (epidermal growth factor) domain-containing protein 1) seems to be localized in the endothelium with developmental functions.

Out of these seven genes, *HSPA4L* and *REEP1* appear to have more relevant functions which might be playing a role for variation in survival against the RGNNV. The involvement of *HSPA4L* with managing stress and cell protection may assist individuals by coping with the pathological condition when the fish is infected with virus. The function of the *REEP1* gene is very relevant to the disease as infected individuals present neurological symptoms with clinical signs characterized as rapid swimming, spiraling, whirling, and lying down at the bottom (Yoshikoshi and Inoue, 1990). The autopsy observations of the VNN disease include hemorrhages in the brain tissues and vacuolization in cells of the spinal cord, brain, and retina along with the high level of necrosis in the nervous cells (Munday et al., 2002; Yang et al., 2021). The findings seem to suggest that the RGNNV mainly targets the nerve cells although lesions can also be detected in the liver and spleen tissues (Yang et al., 2021). The mutations in regulatory regions of a gene may cause its under or overexpression and ultimately deviates in the availability of receptor expression-enhancing protein in the cells. The changes in the availability of protein (abundance or shortage than optimal level) under RGNNV infection might be playing a role for variation in survival against the virus possibly by coping or managing disease symptoms from the hosts. Further studies involving identification/validation of the causative mutation/gene through functional level assays (proteomics, gene editing etc.) would likely assist in unveiling the biological mechanism behind the host resistance against the RGNNV. The selection of individuals based on the actual causative mutation is highly likely to make a faster genetic progress in the desired direction due to increased accuracy of selection compared to when the selection is performed based on markers in LD with the causative mutation.

The accuracy of prediction using validation schemes, random vs. within-family masking did not show significant difference, which may be the result of genetic links across the population as accuracy of predictions using the GS is known to be sensitive to close the genetic relationship between training and candidate populations. Regardless of validation schemes, the average prediction accuracy for survival against VNN using pedigree information was 0.56 while the average accuracy using genomic information was 0.71, which is 29% higher than the pedigree information-based accuracy (Supplementary Table S1.2). The highest gain in accuracy using genomic over pedigree information was 44% which was obtained using the BayesB model (Supplementary Table S1.2). The use of genomic information for survival against VNN clearly outperformed the use of pedigree information-based predictions with a clear advantage. The advantage of genomic information over pedigree mainly contributed through the realized genomic-based relatedness among individuals which deviates from the pedigree information-based relationship coefficients. The realized relationships derived through variations contributed by mutations/variants at the QTL regions possibly become much more important when the QTL(s) have a relatively large effect on the trait. The effect of genomic relationship matrices designed using QTL-linked vs. -unlinked markers on prediction accuracy was recently tested which revealed three times increase in the prediction accuracy with QTL-linked markers compared to when the genomic relationship matrix designed using only unlinked markers (Ling et al., 2021) which highlights the importance of variants within the QTL region. The difference of the prediction accuracy between genomic vs. pedigree is likely to shrink with the availability of deep phenotypes and pedigree information with minimal pedigree errors. The availability of deep pedigree is not possible for the recent commercial populations in breeding, and hence, genomic information can play a very important role to improve traits effectively and efficiently in the desired direction.

The comparison for the accuracy of predictions within genomic models showed that Bayesian models worked better or equally well as GBLUP (Figure 4, Supplementary Table S1.2). The BayesB model outperformed all other genomic models with the highest mean accuracy of 0.76 across the validation schemes (Supplementary Table S1.2). The top ranked BayesB model was followed by BayesC, GBLUP, and lastly Bayesian Lasso with a mean accuracy of ∼0.70 across validation schemes. Hence, the genomic model BayesB showed ∼7% increase in the accuracy compared to other genomic models. The performance of genomic models is known to be affected by the genetic architecture of trait(s), and the Bayesian models are expected to perform better if the trait(s) are affected by a few QTLs (Daetwyler et al., 2012; Van Den Berg et al., 2015; Vallejo et al., 2017). Recently, Aslam et al. (2021) evaluated the accuracy of prediction for survival against the RGNNV using QTL markers (i.e., marker assisted selection, MAS) with the prediction accuracy obtained with the genomic and the pedigree-based models which showed that the gain in the accuracy of prediction using marker-assisted selection was ∼37% higher than pedigree-based selection but fractionally lower than the BayesB model (0.692 with MAS vs. 0.752 with BayesB). The GWAS analysis of our study detected a single genome-wide significant QTL with a large effect and hence validates the expectation on the accuracy of prediction as well supports that the survival against VNN is perhaps affected by a few QTLs.

The presence of a relatively simpler genetic architecture for survival against VNN with a single genome-wide detected QTL explaining a large proportion (though a large part is still missing) of the total genetic variance along with the obtained highest accuracy of prediction using BayesB is very convincing and complimenting results which present a strong potential for the application of efficient and economical marker-assisted selection. Increasing the resolution of a QTL region using more variants (SNPs, deletions, copy number variations, etc.) along with a high-quality phenotype might assist to uncover the missing genetic variance and detect the actual causative mutation affecting the trait.

## Conclusion

The survival against the VNN disease showed low to moderate levels of genetic variation with a possibility of improvement in traits through selective breeding. The genetic architecture for host resistance to the RGNNV appears to be affected by a locus with a large effect and perhaps smaller contribution from the other loci. Multiple genes were identified within the QTL region with the *REEP1* gene located immediately at the upstream of the highest significant SNP which seems to be more pronounced with functions involving the nervous system. The detected QTL explained ∼33% of genetic variance suggesting a strong potential of marker(s)-based efficient and economical selection for improving survival against VNN. The comparative results on the accuracy of predicting breeding values with genomic information were substantially higher (20–44%) than predictions using pedigree information which suggests a strong advantage of using genomics over pedigree-based selection to genetically improve host resistance against the RGNNV.

## Data Availability

The datasets presented in this study can be found in online repositories. The names of the repository/repositories and accession number(s) can be found below: European Variation Archive, accession number PRJEB50333.

## Abbreviations

GBLUP, genomic-based best linear unbiased prediction; GS, genomic selection; GWAS, genome-wide association analysis; h^2^, heritability; HBLUP, hybrid information-based best linear unbiased prediction; LD, linkage disequilibrium; MAF, minor allele frequency; MAS, marker-assisted selection; PBLUP, pedigree-based best linear unbiased prediction; QTL, quantitative trait loci; RGNNV, red-spotted grouper nervous necrosis virus; SNPs, single nucleotide polymorphisms; VER, viral encephalopathy and retinopathy; VNN, viral nervous necrosis; YC, year class.
