# Supplementary material for: Genetic Basis for Resistance Against Viral Nervous Necrosis: GWAS and Potential of Genomic Prediction Explored in Farmed European Sea Bass (Dicentrarchus labrax)
Source: Front Genet. 2022 Mar 25;13:804584. doi: 10.3389/fgene.2022.804584 (PMC8992836; doi:10.3389/fgene.2022.804584)
Supplement: Supplementary file 1 [file DataSheet1.docx]

**Genetic basis for resistance against viral nervous necrosis: GWAS and potential of genomic prediction explored in farmed European sea bass *(Dicentrarchus labrax*)**

*Sergio Vela-Avitúa* ***^1^****, Ingunn Thorland* ***^1^****, Vasileios Bakopoulos* ***^2^****, Kantham Papanna* ***^3^****, Arkadios Dimitroglou* ***^3^****,* *Eleftherios Kottaras* ***^3^****, Papaharisis Leonidas* ***^3^****, Bruno Guinand* ***^4^****, Costas S. Tsigenopoulos* ***^5^****, Muhammad L. Aslam* ***^6^****

***^1^*** *Benchmark Genetics Norway AS (formerly Akvaforsk Genetics Center AS), Auragata 3, NO-6600 Sunndalsøra, Norway.*

***^2^*** *University of The Aegean, Department of Marine Sciences, Laboratory of Ichthyology, Aquaculture and Diseases of Aquatic Animals, University Hill, Mytilene 81100, Greece.*

***^3^*** *Nireus Aquaculture SA., 19400 Koropi, Greece.*

***^4^*** ISEM, Université de Montpellier, CNRS, IRD, EPHE, Montpellier, France*.*

***^5^*** *Institute of Marine Biology, Biotechnology and Aquaculture (IMBBC), Hellenic Centre for Marine Research (HCMR), Heraklion, 71500, Greece*

***^6^*** *Nofima AS, 1430, Norway.*

E-mail addresses:

**SV:** [sergio.vela@bmkgenetics.com](mailto:sergio.vela@bmkgenetics.com)

**IT:** [ingunn.thorland@bmkgenetics.com](mailto:ingunn.thorland@bmkgenetics.com)

**VB:** [v.bakopoulos@marine.aegean.gr](mailto:v.bakopoulos@marine.aegean.gr)

**KP:** [k.papanna@avramar.eu](mailto:k.papanna@nireus.com)

**AD:** [a.dimitroglou@avramar.eu](mailto:a.dimitroglou@nireus.com)

**EK:** [l.kottaras@avramar.eu](mailto:l.kottaras@avramar.eu)

**LP:** [l.papaharisis@avramar.eu](mailto:l.papaharisis@nireus.com)

**BG:** [bruno.guinand@umontpellier.fr](mailto:bruno.guinand@umontpellier.fr)

**CST:** [tsigeno@hcmr.gr](mailto:tsigeno@hcmr.gr)

**MLA**: [luqman.aslam@nofima.no](mailto:luqman.aslam@nofima.no) *

**SUPPLEMENTARY FIGURES**

**Figure S1.1:** The distribution of full sibs per families along with their survival within each family. Each bar represents a full-sib family, and the blue section of each bar indicates the count of surviving individuals while the grey section depicts the dead individuals. The families are sorted based on family size with smallest families at extreme left and the largest families at extreme right. The fluctuation in grey and blue color levels in families present the variation in survival across families.


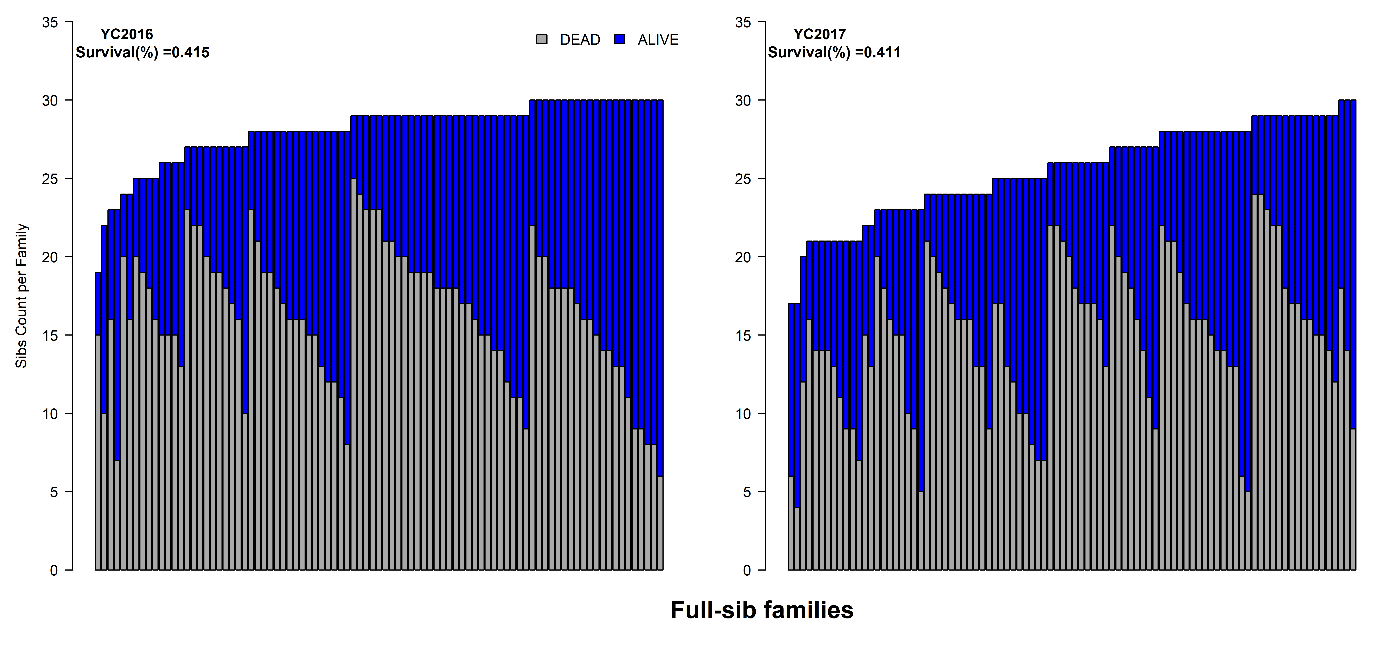


**Figure S1.2:** Manhattan plot with the distribution of $-log10(p)$ values of SNPs across different chromosomes.

The analysis was performed separately for each year-class (YC2016 vs. YC2017) which had gone through the independent challenge tests and plot shows stable QTL signal from both year-classes with even same set of SNPs crossing genome (red) and/or chromosome (blue) wide Bonferroni corrected significance threshold lines.


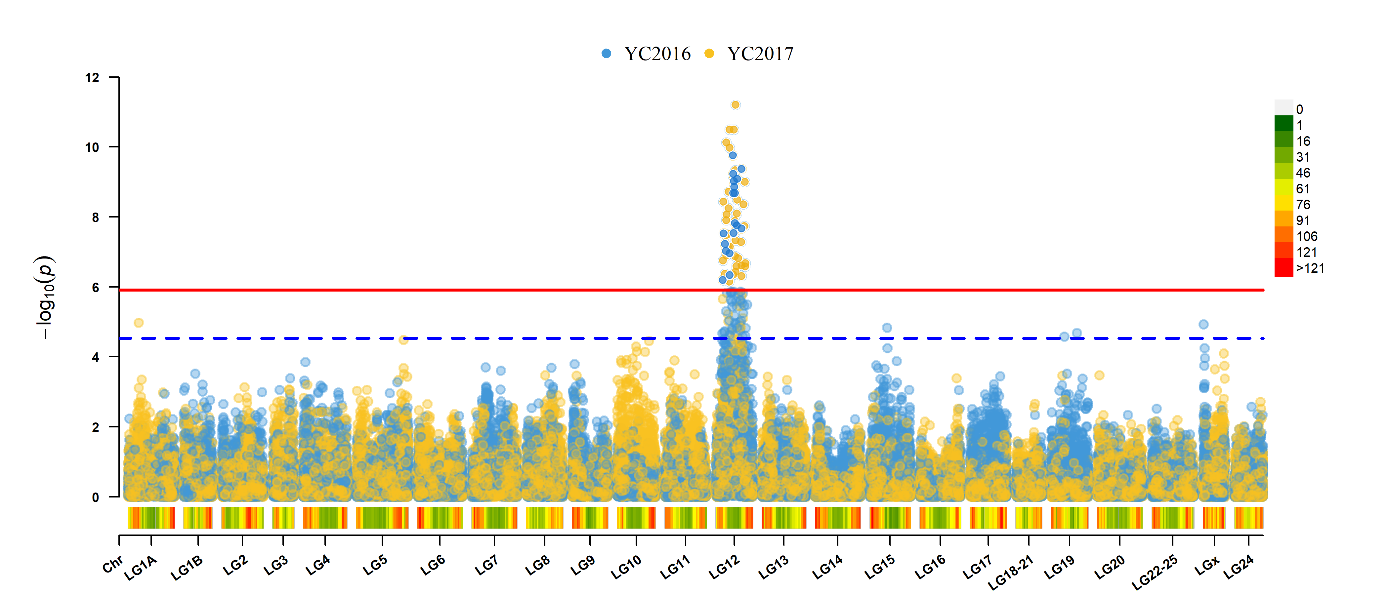


**Figure S1.3:** Manhattan plot with the distribution of $-log10(p)$ values of SNPs across different chromosomes.

**A).** Represents the results when all the markers are a part of genomic relationship matrix with only one fixed effect, batch (YC2016 and YC2017) in the model; **B).** Depicts the results with the highest significant SNP excluded from the G-matrix and used as fixed effect in the model in addition to batch effect.


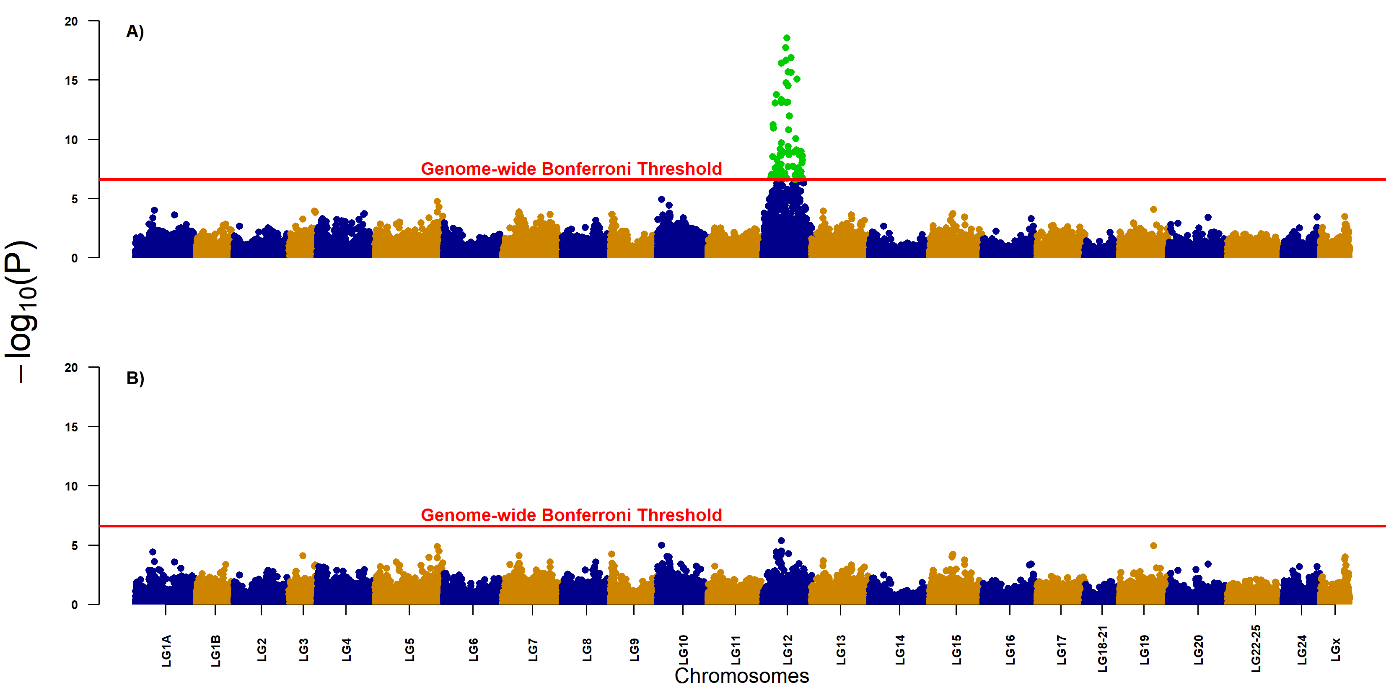


**Figure S1.4:** Quantile-quantile plot with the distribution of observed vs. expected $-log10\left( P-values \right)$.


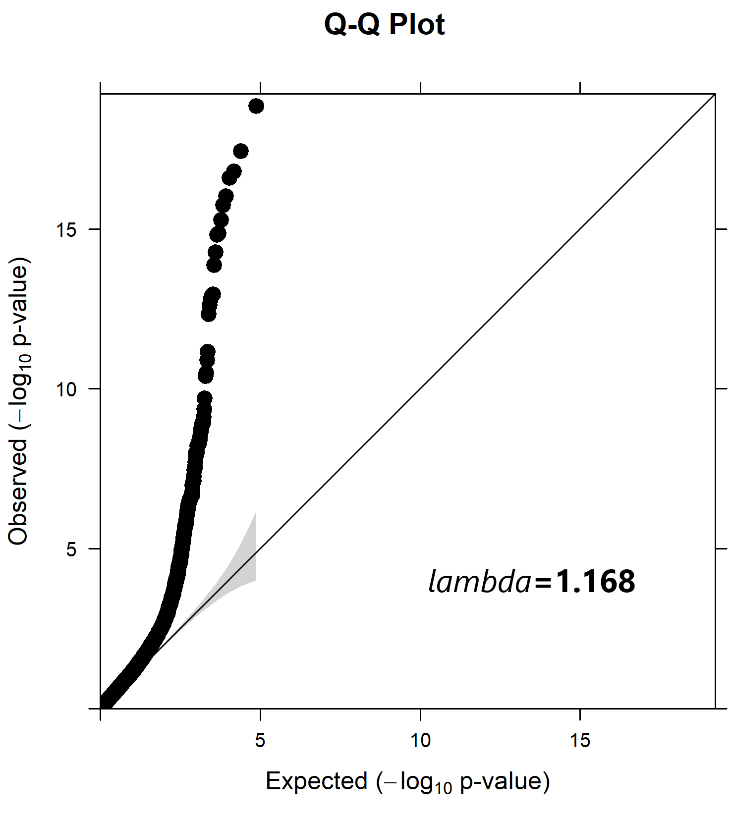


**Figure S1.5:** The principal component analysis-based plot (PCA-plot). **A).**  presenting existence of slight population structure with first principal component explaining ~3.6% while the second component (PCA 2) explain around 2.8% of the variation. **B).** presents the percentage variance explained by all PCAs with the top five PCAs selected for including in GWAS model.


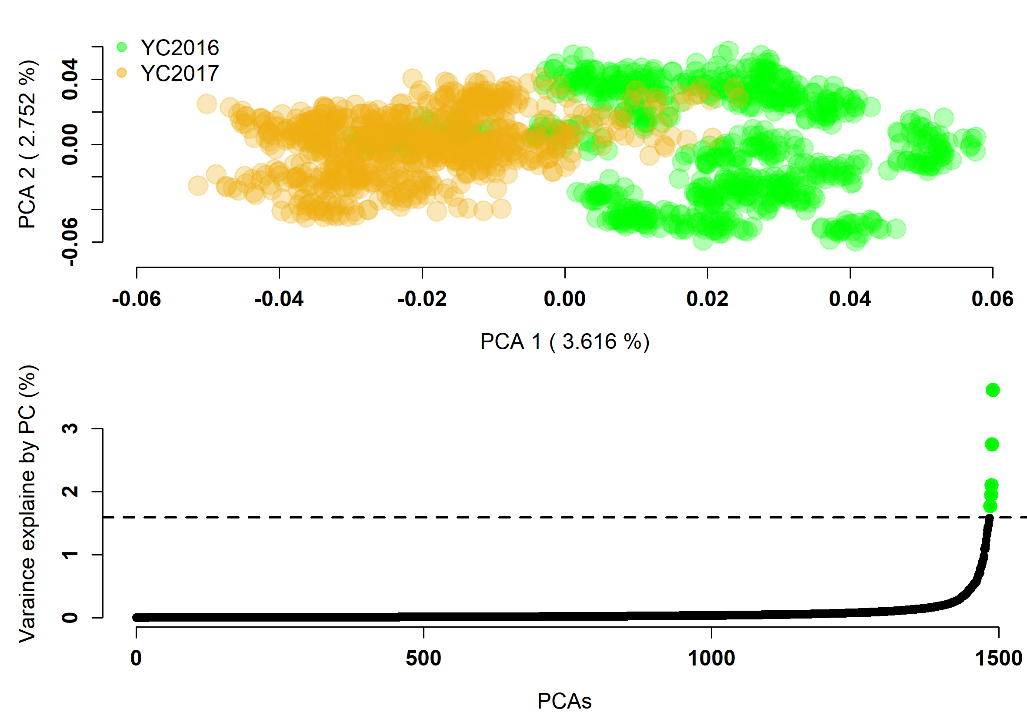


**B)**

**A)**

**Figure S1.6:** The accuracies of prediction for resistance against VNN using PBLUP vs. HBLUP models.


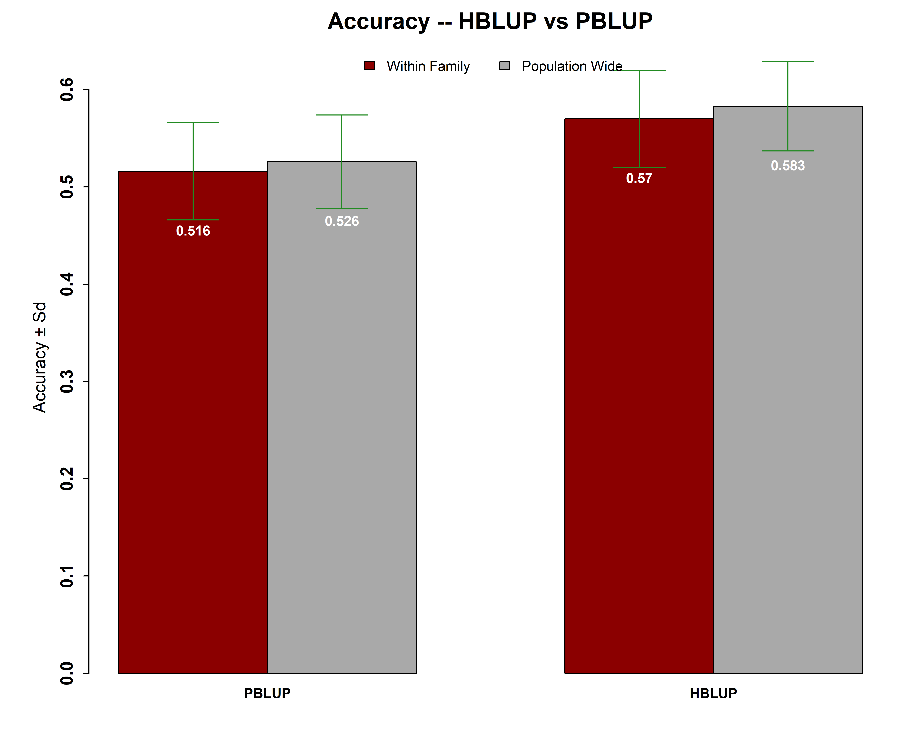


**Figure S1.7:** The distribution of percentage survival and breeding values (GEBV vs. EBV) across the genotypes from the highest significant SNP (“AX-172280857”). **A)** The percentage survival across the genotypes from the highest significant SNP showed ~118% higher survival for the individuals carrying favorable genotype (CC) compared to the individuals carrying homozygous unfavorable (TT) genotype. The number of available surviving individuals under each genotype are highlighted green, showing limited number of individuals available with homozygous favorable genotype (n = 26) which is concordant with the low frequency for the favorable allele (MAF = 0.115) in the population. **B)** The box plot presenting distribution of genomic (GEBV), and pedigree (EBV) based breeding values of individuals across the genotypes of the highest significant SNP. The individuals carrying “CC” genotype showed the highest mean (dark black line in box plot) breeding value followed by heterozygous genotype (“CT”) and the lowest mean breeding value for the individuals carrying “TT” genotype. The overall trend in distribution of GEBV vs. EBV across the genotypes is consistent, however, there is a slightly re-ranking of individuals when ranked with GEBV vs. EBV with a correlation of ~0.80. The red in the plot presenting threshold line for the individuals qualifying 1Sd (standard deviation) above the mean.


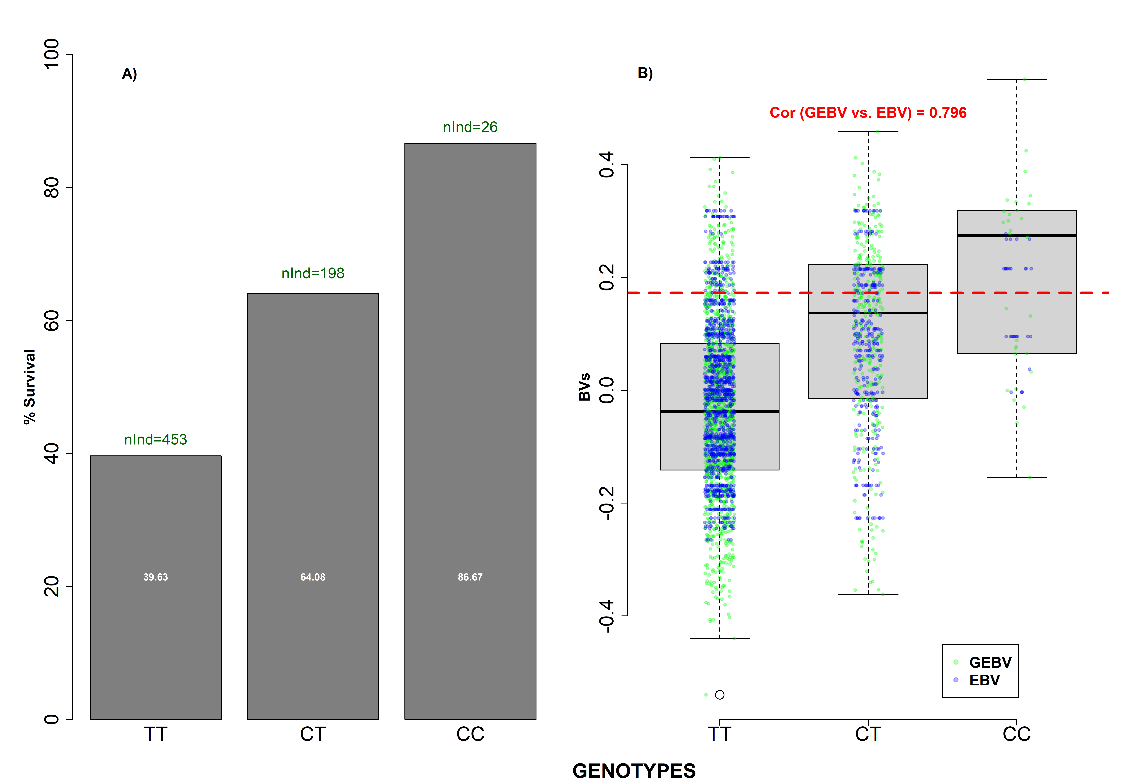


**Figure S1.8:** Heatmap of linkage disequilibrium structure among top 10 genome-wide significant SNPs of GWAS analysis representing mean LD of 0.494. Locus ID with green asterisk sign represents the topmost significant SNP of GWAS.


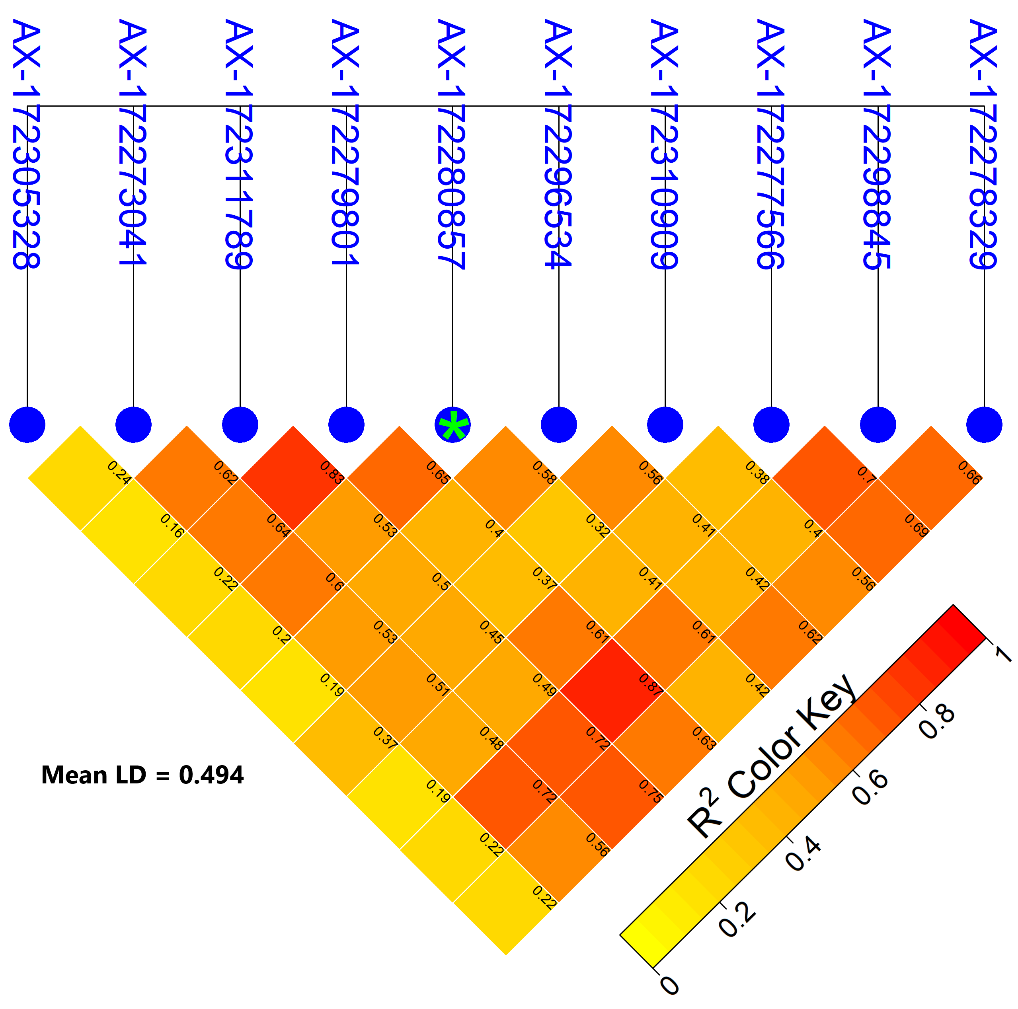


**Figure S1.9:** The genes underlying QTL within ±25Kb region from the highest significant SNP position (SNP: AX-172280857; Position: 11359282).

**SUPPLEMENTARY TABLES**

**Table S1.1:** The reduction of genetic variance with the use of the highest significant SNP as fixed effect in the model.

| **Component/Model** | **M-A1** | **M-A2** |
| --- | --- | --- |
| **Genetic Variance** | 0.06±0.01 | 0.04±0.01 |
| **Residual Variance** | 0.19±0.01 | 0.19±0.01 |
| **Phenotypic Variance** | 0.25±0.01 | 0.24±0.01 |
| **Heritability** | 0.25±0.04 | 0.18±0.04 |

**M-A1**= The estimates from the model when the highest significant SNP was not used as fixed effect; **M-A2** = The estimates obtained with the use of the highest significant SNP as an additional fixed effect in the model. The reduction in the genetic variance due to the addition of the highest significant SNP as fixed effect was 33.33%, which was considered as the proportion of genetic variance contributed from the SNP.

**Table S1.2:** The mean accuracy of predictions with standard deviations acquired from 20 iterations using different models, PBLUP, GBLUP, BayesB, BayesC, Bayesian Lasso.

| **Models/**$\boldsymbol{r}_{\boldsymbol{corr}}$ | **Within Family Masking** | **Random Masking** |
| --- | --- | --- |
| PBLUP | 0.523±0.065 | 0.587±0.108 |
| Bayesian Lasso | 0.686±0.084 | 0.706±0.095 |
| GBLUP | 0.689±0.085 | 0.712±0.094 |
| BayesC | 0.691±0.085 | 0.709±0.092 |
| BayesB | 0.751±0.094 | 0.767±0.093 |

Within family masking refers to the validation scheme **“a”** where masking for 30% of the individuals was performed with each family while random masking refers to the validation scheme **“b”** where random masking of 30% of the individuals was performed regardless of family.

**Table S1.3:** The information on genes underlying 50Kb region of the QTL.

| **CHR** | **Gene_Start** | **Gene_End** | **Description** |
| --- | --- | --- | --- |
| LG12 | 11339327 | 11339425 | Inositol-tetrakisphosphate 1-kinase-like, ***ITPK1*** |
| LG12 | 11345684 | 11345792 | Serine threonine-protein kinase plk4 isoform 1, ***PLK4*** |
| LG12 | 11354293 | 11354478 | Heat shock 70 kda protein 4l, ***HSPA4L*** |
| LG12 | 11370762 | 11370829 | Receptor expression-enhancing protein 1-like, ***REEP1*** |
| LG12 | 11364670 | 11364714 | Charged multivesicular body protein, ***CHMP2*** |
| LG12 | 11377257 | 11377426 | 39s ribosomal protein mitochondrial precursor, ***MRPL35*** |
| LG12 | 11381469 | 11381623 | Scavenger receptor class f member 1-like isoform x1, ***SCUBE1*** |
